# Arbuscular mycorrhizal fungi promote the growth of plants in the mining associated clay

**DOI:** 10.1038/s41598-020-59447-9

**Published:** 2020-02-14

**Authors:** Ziheng Song, Yinli Bi, Jian Zhang, Yunli Gong, Huihui Yang

**Affiliations:** State Key Laboratory of Coal Resources and Safe Mining, College of Geoscience and Surveying Engineering, China University of Mining and Technology (Beijing), Beijing, 100083 P.R. China

**Keywords:** Environmental sciences, Environmental impact

## Abstract

It is urgent to restore the ecological function in open-pit mining areas on grassland in Eastern China. The open-pit mines have abundant of mining associated clay, which is desirable for using as a soil source for ecological restoration. The mining associated clay in Hulunbuir district, Inner Mongolia was selected and mixed with a sandy soil at a ratio of 1:1 (S_C soil). Also, effects of arbuscular mycorrhizal fungi (AMF) inoculation on soil functions were studied. The aboveground and underground biomass of maize in S_C soil was 1.49 and 2.41 times higher than that of clay soil, respectively. In the topsoil and S_C soil, the growth hormone (IAA) and cytokinin (CTK) levels of maize were higher than that of clay, while abscission acid (ABA) levels were lower. The inoculation with AMF could significantly improve the biomass of maize and enhance the stress resistance of plants. Through structural equation model (SEM) analyses, it was found that the soil type and AMF inoculation had the most direct impact on maize growth and biomass content. These finds extend our knowledge regarding a low-cost method for physical and biological improvement of mining associated clay, and to provide theoretical support for large-scale application in the future.

## Introduction

Coal is one of the main energy sources in China, but coal mining can be harmful to environment, especially the open-pit mining has a direct impact on the environment^1–3^. The dump soil area generated by opencast mining leads to serious disturbance of soil layer structure^4^. Open-pit stripped materials contain thick associated clay layers, which can be utilized as resources to alleviate the problem of surface soil barrenness in mining areas. Anikwe^5^ found that adding rice husks into soil improved the clay permeability, soil aeration, and microbial activity. It was proposed that mixing sand and clay can effectively reduce soil compaction and improve the nutrient availability of clay particles^6^. Tahir^7^ found that microorganisms inoculated in soil were more likely to adhere to clay particles, thus efficiently and sustainably promoting soil functions. The associated clay has high content of organic matters and strong capacity of water-holding. However, it has low nutrient availability, thus it is not suitable for crop planting.

Arbuscular mycorrhizal fungi (AMF) are soil microorganisms, which are capable of forming potential symbiotic relationships with the roots of host plants^8,9^. Mycorrhizal mycelium can enhance the absorption range of roots and improve the resistance of plants to external stress^8,10–13^, especially in the environment of high-pressure open-pit mines, they can promote plant growth and improve the vegetation recovery in the mine reclamation area^14^. Compared with inoculation of arbuscular mycorrhizal fungi (AMF) into topsoil, inoculation of AMF into clay can better promote plant growth and increase nutrient availability^15,16^. As a biological modifier, AMF can effectively improve soil structure and promote plant growth under both normal and stressed conditions^17,18^. AMF can also affect the root morphology of plants, enhance the ability of root branching, expand the absorption range of root, and thus increase the absorption of water and nutrients^19,20^. In addition, AMF can effectively increase the absorption and utilization of mineral nutrients by plants^21,22^.

Excessive clay content in mining-associated clay can inhibit plant growth, and the stress of clay on plants can be reduced by changing soil properties. The stress resistance of plants can be increased by inoculating AMF. In order to make efficient use of the associated clay in the open-pit mining, this study adopted a 1:1 ratio of sand and inoculated mycorrhizal to solve problems of low survival rate of plants and inability to directly utilize associated clay in open pit dump construction. This study intended to develop a low-cost method for physical and biological improvement of mining associated clay, and to provide theoretical support for large-scale application and promotion in the future.

## Materials and Methods

### Soil and materials

**Figure 1:**
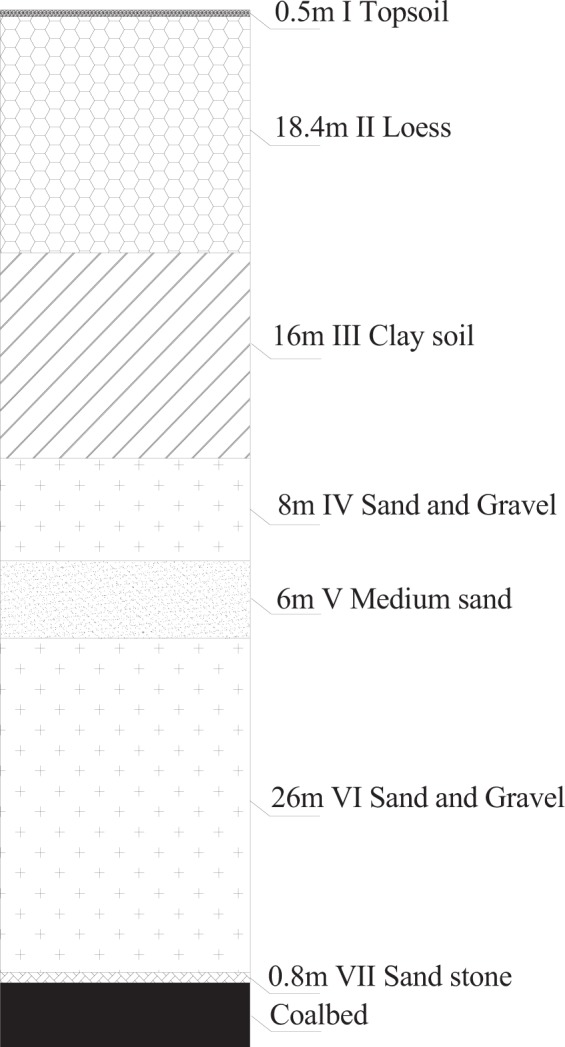
Geologic profile of open-pit mines in HulunBuir grasslands of Inner Mongolia, northeast China.

The clay used in the experiment was collected from an open-pit mine in a grassland of Eastern China (Hulunbuir district, Inner Mongolia). The study area contains seven layers of overlying rock and soil. The geological profile is shown in Fig. 1. The first layer (layer I in Fig. 1) is topsoil with a maximum thickness of 0.5 m. The second layer (layer II) is loess with a thickness of 18.4 m. The third layer (layer III) is clay with a thickness of 16 m. The fourth layer (layer IV) is sandy gravel layer with a thickness of 8 m. The fifth layer (layer V) is medium sand with a thickness of 6 m. The sixth layer (layer VI) is sand gravel with a thickness of 26 m. The seventh layer (layer VII) is sandstone with a thickness of 0.8 m. Since the layers below layer III are composed of hard rocks which are not suitable for studying as surface soil, only Layer III (Clay) was chosen as the research object for analyzing its feasibility as a substitute material for topsoil. The basic index of soil is listed in Table 1. The sandy soil used in the experiment was collected from a river near the open-pit mine.

**Table 1:** Selected initial physico-chemical properties of sandy soil, clay soil, and top soil used in this study.

soil	pH	EC (μS cm^−1^)	Particle size			Water holding capacity(%)	Available N (mg kg^−1^)	Available P (mg kg^−1^)	bacteria	fungus
			Sand (%)	Silt (%)	Clay (%)				cfu g^−1^	
Top soil	7.52	237	35.5	21.7	42.8	35.23	124.37	13.71	5.4 × 10^5^	1700
Clay soil	7.8	287	0	1.9	98.1	46.54	86.9	17.04	200	0
Sandy soil	7.23	0.08	97.6	2.4	0	23.51	14	4.95	3.2 × 10^5^	100

### Experimental design

The air-dried topsoil, clay soil, and sandy soil were sifted through a 2 mm sieve to remove plant residues and large particulate matter. Four soil types were used: topsoil, clay soil, sandy soil, and a mixture of clay soil and sandy soil at a ratio of 1:1 (W/W, denoted as S_C soil) (Table 2). In order to reduce the influence of soil indigenous soil borne microorganisms in different soils on the experimental results, all soils were sterilized at 121 °C and 100 kPa for 2 h At the beginning of the experiment, a mixed matrix was evenly stirred and packed into a plastic pot with a dimension of 19 cm (height)*20 cm (diameter of pot mouth)*16 cm (diameter of pot bottom), 5 kg for each basin. At the same time, inoculation (M) and non-inoculation (CK) were designed for each soil substrate. A total of 8 treatments, each with three replicates, were carried out in a sunlight greenhouse in the Microbial Reclamation Laboratory of China University of Mining and Technology (Beijing) with an average diurnal temperature range of 18–30 °C.

**Table 2:** Experimental Design.

Treatment	Soil treatment	AMF	Denoted
M	Top soil	*F. mosseae* inoculum	Top soil + M
	Clay soil		Clay soil + M
	Sandy soil		Sandy soil + M
	Clay soil + sandy soil (1:1 W/W)		S_C soil + M
CK	Top soil	sterilized *F. mosseae* inoculum	Top soil
	Clay soil		Clay soil
	Sandy soil		Sandy soil
	Clay soil + sandy soil (1:1 W/W)		S_C soil

For using the soils for planting, full-grained maize seeds (variety Pinnuo 28) were selected. They were soaked in a solution of 10% H_2_O_2_ for 10 minutes and incubated at 25 °C for 24 h. Then they were sowed in a pot and buried in a hole with a depth of 3–5 cm (1 seed per hole, 3 seeds per pot). Before planting, the soil was watered to the maximum water holding capacity. After 24 hours of natural water balance, nutrient solution was added to adjust the concentration of N and P to 150 g/kg and 45 g/kg, respectively. The AMF inoculum, provided by the Institute of Plant Nutrition and Resources, Beijing Academy of Agriculture and Forestry Sciences, consisted of spores (1610 spores per 100 g of soil), external mycelium, and mycorrhizal root fragments (90% mycorrhizal colonization rate). The seeds in the AM treatment were simultaneously given 50 g of *Funneliformis mosseae (F. mosseae*) inoculum per hole, and the seeds in the CK treatment were given 50 g of sterilized *F. mosseae* inoculum per hole. Only one plant per hole remained at the three-leaf stage. After sowing, the plant was placed in a greenhouse for cultivation with natural light. Only one plant per pot remained at the trifoliate stage. The water content was adjusted to 60% of the maximum moisture content every 5 days, and the planting time is 60 days.

### Determination of Indicators and methods

Maize was harvested after 60 days of planting. The abundance of AMF hyphae in the soil is typically measured using the modification by Miller *et al*.^23^, briefly, extracted the hyphae from a 5 g soil of each sample by stirring it into 90 mL of 20 g/L sodium hexametaphosphate on a stir plate, suction filtering subsample through filter paper (1.2 µm mesh), staining overnight with trypan blue, and quantifying AMF hyphal abundance using the gridline intercept method at 400x magnification. Maize biomass and plant hormones were calculated. Endogenous plant hormones IAA, CTK, ABA were determined by ELISA with enzyme-labeled kit produced by the Shanghai-based Immunity Company. Specific methods are described in Bi *et al*.^24^.

Fifteen root segments were randomly (1.5 cm length) selected in each treatment and stained with 0.05% trypan blue^25^. The equation of mycorrhizal colonization rate is as follows.

1 $${\rm{MCR}}={\rm{MR}}/{\rm{TR}}\times 100 \% $$

where MCR is mycorrhizal colonization rate, MR is the number of mycorrhizal root segments, TR is the total number of root segments.

The mycorrhizal responsiveness was defined as improvement of biomass in this study. The equation of percentage increase in plant biomass in response to mycorrhizal colonization is as follows.

2 $${\rm{MR}}=({\rm{TBI}}-{\rm{TBN}})/{\rm{TBI}}\times 100 \% $$

where MR is the mycorrhizal responsiveness, TBI is the total biomass of inoculated maize, TBN is the total biomass of non-inoculated maize.

Soil samples were collected from the top 10 cm of the profile with a spiral drill (diameter 35 mm). Three soil cores were collected from each pot and combined into one sample. Plant residues in the soil were separated with hands. After separation, soil samples were placed in plastic bags for analyzing soil enzyme activity before being stored at 4 °C. A method described by Wu *et al*.^26^ was used to determine the soil urease activity. Briefly, soil samples were incubated with 10% urea solution at pH 7.1 at 37 °C for 24 h, and then phenol sodium hypochlorite solution was added. After proper dilution using ultrapure water, quantitative analysis of ammonium concentration was performed with a spectrophotometer (Leng Guang Tech 752sp, CHN) by reading the absorbance at 578 nm. The activity of acid phosphatase in soils was determined by a method developed by Tabatabai^27^. The pH of acid phosphatase was 6.5, the pNP(p-nitrophenol) released from phosphatase was colorimetrically determined by reading the absorbance at the wavelength of 400 nm, and the enzyme activity was expressed as the amount of p-nitrophenol produced per gram of soil.

### Statistical analysis

In this study, SAS 9.0 statistical software was used to analyze the variance of the experimental data, with a significant level of 5%. Drawing was conducted by using R^28^.

## Results

### Effects of AMF on plant growth in different soils

The amount of biomass in different soils was in a range of 7.76 g to 16.42 g, with Clay Soil being the lowest at 7.76 g, while S_C Soil-M being the highest, at 16.42 g (Table 3). The order of both aboveground biomass and underground biomass was S_C soil > Top soil > Sandy soil > Clay soil. The contents of biomass in Top soil and S_C soil were significantly higher than that in Clay soil and Sandy soil (P < 0.05). The aboveground and underground biomass in S_C soil were 1.49 and 2.41 times of that in Clay soil, respectively. After inoculation, the mycelium infection rate was higher than 80%, and the mycelium density in Clay soil + M was the lowest at 1.54 m/g, which was significantly lower than that in Top soil + M, Sandy soil + M, and S_C soil + M. The inoculation significantly increased the amount of biomass in Top soil, S_C soil, and Sandy soil, and the above-ground part increased by 28.3%, 34.8%, and 24.4%, respectively, while the underground part increased by 37.3%, 20.6%, and 34.8%, respectively. The highest mycorrhizal responsiveness was 34.83% in S_C soil, followed by 29.45% in Top soil. There was no notable increase of maize biomass by inoculation in Clay soil, and the mycorrhizal responsiveness in Clay soil was only 11.85%.

**Table 3:** Effects of different treatments on plant biomass.

	Mycorrhizal infection rate %	Mycelium density m g^−1^	above-ground biomass g plant^−1^	underground biomass g plant^−1^	Mycorrhizal responsiveness %
Top soil	0	0	11.07 ± 1.1b	1.58 ± 0.04b	29.45
Top soil + M	83 ± 3a	3.61 ± 0.11a	14.2 ± 0.22a	2.17 ± 0.13a	
Sandy soil	0	0	8.17 ± 0.27 cd	0.94 ± 0.13 cd	25.48
Sandy soil + M	83 ± 3a	3.88 ± 0.09a	10.16 ± 0.84bc	1.27 ± 0.07c	
Clay soil	0	0	7.08 ± 0.59d	0.7 ± 0.06d	11.85
Clay soil + M	80 ± 2a	1.54 ± 0.21b	7.86 ± 0.34bcd	0.82 ± 0.05c	
S_C soil	0	0	10.57 ± 0.45b	1.61 ± 0.07b	34.83
S_C soil + M	83 ± 3a	3.79 ± 0.13a	14.25 ± 0.8a	2.17 ± 0.14a	

Values are means ± standard deviation of triplicate measurements. Mean values with the same letter are not significantly different among treatments at the 5% level.

### Effect of AMF on soil enzyme activity

Urease produces ammonia as one of the nitrogen sources for plants, and it can be used to characterize the nitrogen status of soil. For evaluating the strength of phosphorus bioconversion in soil, phosphatase activity can be used as an index, since acid phosphatase can accelerate the dephosphorization of organophosphorus.

**Figure 2:**
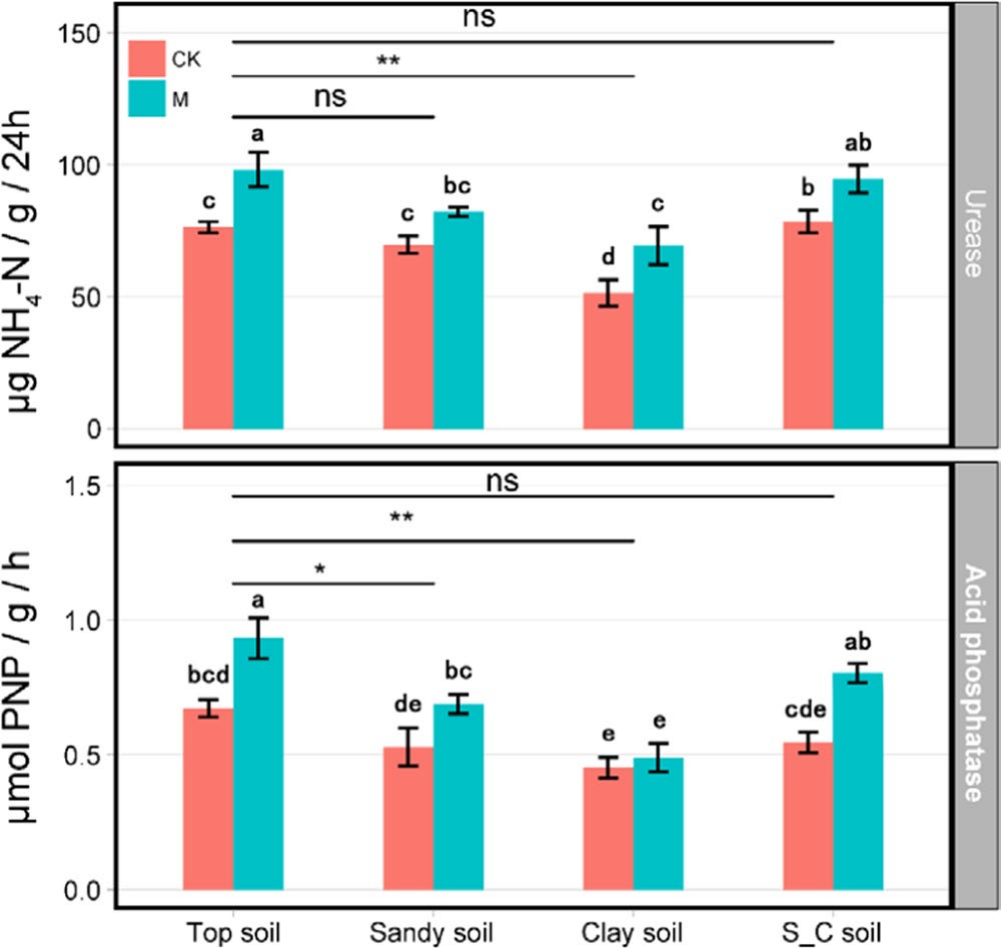
Soil enzyme activities of different soil types and treatments. Values are means ± standard deviations of triplicate measurements. Mean values with the same letter are not significantly different among treatments at the 5% level.

The activities of urease and acid phosphatase in S_C soil were not significantly different from that in Top soil (Fig. 2). While the activities of urease and acid phosphatase in Clay soil was significantly lower than that in Top soil. The content of urease in Sandy soil was not significantly different from that in Top soil, but the content of acid phosphatase was significantly lower than that in Top soil. The lowest urease activity was 51.48 μg NH_4_-N/g/24 h in Sandy Soil treatment and 98.14 μg NH_4_-N/g/24 h in Top soil-M treatment. In CK groups, the order of urease activity in soil was Top soil > S_C soil > Sandy soil > Clay soil. The urease activities of Top soil, S_C soil, and Sandy soil were significantly higher than that of Clay soil. After inoculation, urease activities of Top soil, Clay soil, and S_C soil were significantly higher than those in non-inoculated soil. The urease activities of Top soil and S_C soil increased by 28.6% and 20.56% after inoculation. The lowest acid phosphatase activity was 0.45 mol PNP/g/h while the highest was 0.93 mol PNP/g/h for Top Soil -M. There was no significant difference in the activity of acid phosphatase between different soil types, but all soil types were significantly higher than that of Sandy soil (P < 0.05). Similar to the soil urease activity, the acid phosphatase activity in Clay soil was significantly lower than that in other soil types. Compared with non-inoculation, acid phosphatase activity in Top soil, S_C soil, and Sandy soil was significantly increased by inoculation, which was 38.6%, 46.9%, and 30.2%, respectively. However, there was no notable change of acid phosphatase activity in Clay soil, which only increased by 8% after inoculation.

### Effects of AMF on plant hormone levels in different treatments

**Figure 3:**
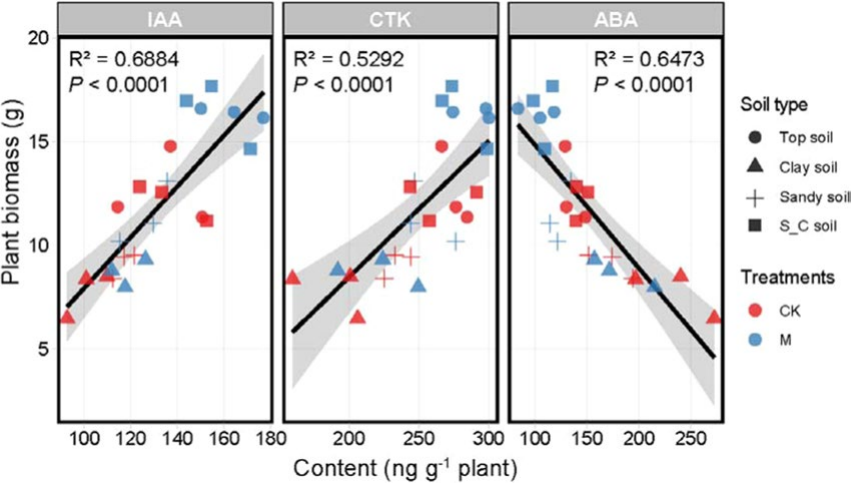
Plant Hormone Levels in Different Treatments of different soil types and treatments.

Compared with three endogenous hormone levels (Fig. 3) in maize roots, the different soil types have significant effects on IAA, CTK and ABA. Plant biomass was positively correlated with IAA and CTK, and negatively correlated with ABA. Top soil and S_C soil had the highest IAA, CTK content and the lowest ABA content. Top soil and S_C soil had 1.32 times and 1.35 times of IAA content as many as Clay soil. CTK also had a similar pattern, showing that Top soil and S_C soil had higher contents of CTK than Sandy soil and Clay soil. In Top soil, the contents of IAA and CTK after inoculation increased by 22.2% and 5.4%, respectively, while ABA was inhibited by 24.7% after inoculation.

### Multiple factors accounting for plant biomass

**Figure 4:**
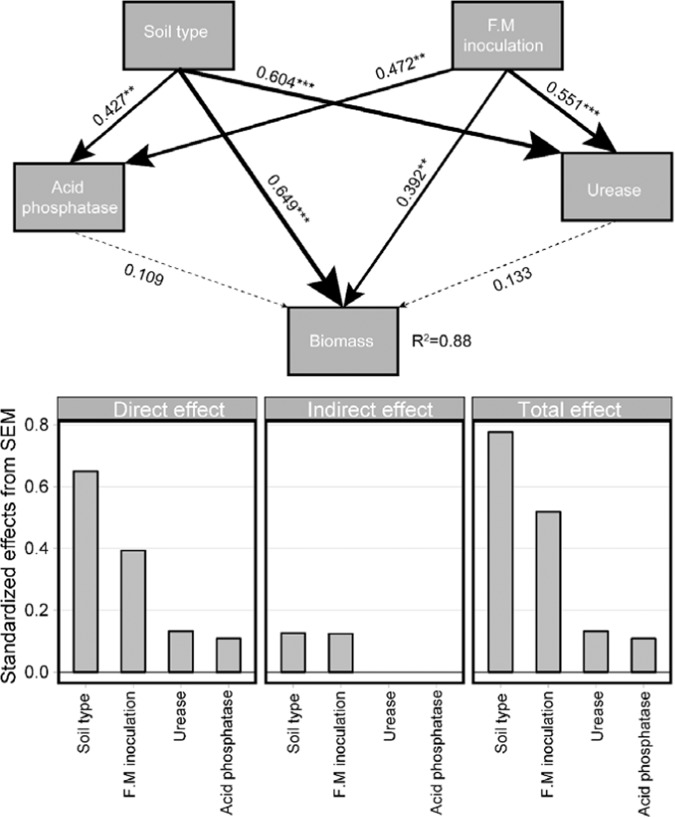
A structural equation model showing the direct and indirect effects of soil type, treatment, acid phosphatase, and urease on the plant biomass. Solid and dashed arrows indicate significant and nonsignificant relationships, respectively. The width of the arrows is proportional to the strength of the path. R^2^ denotes the proportion of variance. Standardized effects (total, direct, and indirect effects) are derived from the structural equation model. The hypothetical model fits our data well: χ2 = 1.37, P = 0.50, df = 2, GFI = 0.98, and RMSEA = 0.00.

We established a structural equation model (SEM) to evaluate the direct and indirect effects of multiple drivers on plant biomass (Fig. 4). An important function of SEM is to partition the direct and indirect effects that variables might have on each other. Our model could explain 88% of the variation in plant biomass. Soil type and inoculation of *F. mosseae* could directly affect the biomass, and also indirectly affect it by affecting the activities of urease and phosphatase. Soil type and inoculation of *F. mosseae* had direct effects on biomass. Another important function of SEM is to assess the intensity of the (direct and indirect) effects of these multi-drivers. We calculated the standardized direct, indirect, and total effects. In terms of total effects, soil type was the most important positive factor affecting plant biomass, followed by *F. mosseae* inoculation.

## Discussion

### Improving mining associated clay properties by mixing sand soil

Due to the urgent need of soil reconstruction in the special environment of open-pit mines, it is important to use the associated clay as a topsoil replacement. Enzymes activities are considered as important “sensors” for microbial population and for soil chemical and physical conditions^29^. The importance of microbial enzymes in an ecosystem has been extensively demonstrated and therefore microbial enzymes activities have been used to test the effects of different treatments on soil quality^30–33^. The improved enzyme activity of soils is largely due to the increasing of organic matter in the soils. For example, Akhtar *et al*.^34^ found that the activities of urease and phosphatase in the soil increased significantly after covering the silty clay with straw, because the straw mulching became a substrate for soil enzymes^35^. Innangi *et al*.^36^ found that the soil urease activity increased during the mineralization of organic matter by using olive fruit residue to improve the soil. However, it is not practical to use organic matter for improving soils at a large-scale soil reconstruction in areas with open-pit dumps. In this current study, we found that the soil urease and acid phosphatase activities can be increased by mixing a widely available sandy clay with pure clay, urease activities in S_C soil is 1.52 times and 1.12 times of that in clay soil and sandy soil, respectively. The activity of phosphatase shows a similar pattern, which is S_C soil higher than clay soil and sandy soil. Urease activity is highest in top soil, then in S_C soil, which is in agreement with previous studies, because the topsoil contained the highest content of organic matters compared with other soil types. The acid phosphatase in clay is 0.45 μmol PNP/g/h, which lower than that in other treatments, which is related to the low biomass of underground part of corn, because the enhancement of rhizosphere acid phosphatase activity can be directly produced by plant roots or indirectly by stimulating microorganisms^37^. Water content also has an effect on soil enzyme activities. Zhang *et al*.^38^ reported that urease and phosphatase activities in alternate wetting and drying environments with relative drying conditions are higher than those in continuous immersion conditions. In the current study, the yield of corn in clay was the lowest. Although clay has excellent water-holding and nutrient-storage capacities, it will lead to difficulties in transporting water and nutrient when its content is too high, resulting in the poor development of root system. As a result, we adjusted the clay to 60% of its maximum water holding capacity. For the S_C soil, it had a good balance between water retention and nutrient transportation, so urease and acid phosphatase activities in S_C soil were higher than that in clay or sand.

### Improving mining associated clay matrix by inoculating AMF

Taher^39^ investigated the growth of plants with AMF in tailings of coal mining areas. They found that plants could grow larger through the interaction with fungal communities regardless of soil types. Our current study also found that AMF can effectively increase the plant biomass when water content decreased and soil type changed. In addition, Taher^14^ found that the growth rate of trees in mining area was significantly slower than that in natural area, and colonizing trees with AMF could enhance the adaptability of trees to the mining environment. Also, AMF might promote plant growth in the case of drought or soil compaction

Inoculation can improve the enzyme activity in soil and alleviate the stress of mining associated clay, this is consistent with our research results, after AMF inoculation, urease activity in Top soil, Sandy soil, S_C soil and Clay soil increased by 28.6%, 17.8%, 34.6% and 20.6% respectively. AMF is interrelated with most plant speciation^40^, the contribution rates of mycorrhiza in Top soil, Sandy soil, Clay soil, S_C soil are 29.45%, 25.48%, 11.85%, 34.83%, respectively, so it can be a key factor in the ecological reclamation of mining areas. AMF improves the effectiveness of P and N^41^, as well as other nutrients such as micronutrientsfor plant growth, and also promotes the absorption of fertilizer by plants^42^, this is also the same as our results, the biomass of plant roots increased by 17~37% after AMF inoculation. In this study, we found that AMF inoculation could improve the biomass of plants with varied degree of improvement at different soil conditions. The biomass of maize in pure clay was the lowest. Although the biomass in pure clay was significantly improved by AMF inoculation, the mycelium density and biomass in pure clay were lower than those in other soil types. It might be ascribed to the limited spread of mycelium in clay. This study may suffer the following limitations because we used the autoclaved soil is to mitigate the impacts of soil indigenous soil borne microorganisms to get a causal relationship between adding Arbuscular mycorrhizal fungi (*F. mosseae*) and plant growth. While the autoclave process may also alter soil physiochemical properties, thus we adjusted the soil nutrients to the same level before carried out plant growth experiments to minimize the potential impacts. Nevertheless, we suggested more filed studies were needed to give more a robust evidence. However, in other soil types, mycelia could spread well and increase the adsorption of nutrients by plants.

### Effects of different treatments on physiological and biochemical characteristics of plant growth

Seleiman *et al*.^43^ planted corns in silty clay (silty clay 41.9%, clay 42.5%, fluvial, lacustrine) and sandy soil (silty clay 4.6%, clay 6.8%, marine), and found that silty clay produced a high yield of maize. The mining associated clay used in this current study was a kind of heavy clay (silt content 0%, clay content 98.1%), and the yield was even lower than that of sandy soil. Therefore, we hypothesized that the high clay content in clay soil could cause stress on plant growth, resulting in a decline in yield. To confirm our hypothesis, we analyzed the plant hormones. Plant hormones (plant growth regulators) are compounds that function at very low concentrations to regulate various cellular processes and plant responses to changes in environment^44,45^. Among them, ABA is the key hormone that regulates the response of plants to abiotic stress^46^. The IAA content of maize in Clay soil is 75.37% of that in Top soil, indicating that plant growth is under stress, and IAA and CTK have been considered to be related to plant yield and productivity^47,48^. Our results are consistent with previous studies. The level of IAA and CTK in topsoil and S_C treatment was higher than that in clay and sand treatment. Meanwhile, the biomass of maize in Top soil was 1.62 times of that in Clay soil, and that of S_C soil was 1.56 times of that in Clay soil. Some studies have suggested that in the case of insufficient water in plants, cell dehydration leads to a decrease in IAA level^49^ and an increase in ABA^50^. Therefore, the levels of IAA and CTK hormones in sandy soil will be reduced and ABA content will be increased. In this study, we found that that with time clay was drying out and cracking. No cracking were found in the top soil and the S_C treatment.

In this current study, we found that AMF inoculation could alleviate plant hormone imbalance caused by different soil types, by significantly increase the levels of IAA and CTK, reduce the ABA level of maize roots, and significantly increase the biomass of maize. This result is in agreement with previous reports that inoculation with AMF has a tendency to increase plant hormone levels (auxin, cytokinin)^51^, and the IAA, CTK, and other plant hormones released by AMF inoculation can contribute to plant growth^39^. In our study, we found that the IAA of plants in clay increased by 17.26% after AMF inoculation, indicating that AMF inoculation can alleviate this stress and promote the growth of plants at the same time. In addition, AMF not only provides mineral nutrition for plants to exchange carbon assimilates, but also alters the homeostasis of plant hormones. Based on results from previous studies by others and our current results, we believe that AMF can alleviate plant stress and promote plant growth.

## Conclusion

Our study represents the first attempt to improve the mining associated clay in grassland open-pit mines in eastern China. We found that mixing sandy soil with clay could promote the activity of soil enzymes and significantly increase the yield of maize compared with pure clay. SEM analysis showed that the main factor affecting maize biomass was soil type, followed by inoculation of *F. mosseae*. After inoculation with AMF, the yield of maize was increased, the stress resistance of maize was enhanced, and also increased the activity of soil enzymes. AMF could also act as biological improvers in mining associated clay. The excessive clay content in clay resulted in stress on plant growth, decrease of soil enzyme activity, decrease of IAA and CTK levels, increase of ABA levels, and lowered the biomass of both above-ground and underground parts of plants. Mixing clay and sandy soil at a ratio of 1:1 could alleviate the stress on plants and increase the plant biomass and soil enzyme activities to a level similar to that of top soil. We believe that mixing mining associated clay soil with sandy soil can effectively improve the mining associated clay soil quality. And AMF inoculation can further promote plant growth.
