# Author Correction: Arbuscular mycorrhizal fungi promote the growth of plants in the mining associated clay

**DOI:** 10.1038/s41598-020-75521-8

**Published:** 2020-10-22

**Authors:** Ziheng Song, Yinli Bi, Jian Zhang, Yunli Gong, Huihui Yang

**Affiliations:** State Key Laboratory of Coal Resources and Safe Mining, College of Geoscience and Surveying Engineering, China University of Mining and Technology (Beijing), Beijing, 100083 P.R. China

Correction to: *Scientific Reports* https://doi.org/10.1038/s41598-020-59447-9, published online 14 February 2020

In the original version of this Article, Z. Song and Y. Bi were omitted as equally contributing authors.

Additionally, the original version of this Article contained an error in the Author Contributions section.

“Z. Song and Y. Bi designed experiments; Z. Song, J. Zhang and H. Yang collect soil samples; Z. Song, J. Zhang and Y. Gong carried out experiments; Z. Song and J. Zhang analyzed experimental results. Z. Song and Y. Bi wrote the manuscript.”

now reads:

“Z. Song and Y. Bi contributed equally to this work. Z. Song and Y. Bi designed experiments; Z. Song, J. Zhang and H. Yang collected soil samples; Z. Song, J. Zhang and Y. Gong carried out experiments; Z. Song and J. Zhang analyzed experimental results. Z. Song and Y. Bi wrote the manuscript.”

These errors have now been corrected in the PDF and HTML versions of the Article.

